# Testing Nonlinearity with Rényi and Tsallis Mutual Information with an Application in the EKC Hypothesis

**DOI:** 10.3390/e25010079

**Published:** 2022-12-31

**Authors:** Elif Tuna, Atıf Evren, Erhan Ustaoğlu, Büşra Şahin, Zehra Zeynep Şahinbaşoğlu

**Affiliations:** 1 Department of Statistics, Faculty of Sciences and Literature, Yildiz Technical University, Davutpasa, Esenler, 34210 Istanbul, Turkey; 2 Department of Informatics, Faculty of Management, Marmara University, Göztepe, 34180 Istanbul, Turkey; 3 Department of Computer, Faculty of Engineering, Halic University, Eyupsultan, 34060 Istanbul, Turkey

**Keywords:** nonlinearity, Rényi mutual information, Tsallis mutual information, EKC hypothesis

## Abstract

The nature of dependence between random variables has always been the subject of many statistical problems for over a century. Yet today, there is a great deal of research on this topic, especially focusing on the analysis of nonlinearity. Shannon mutual information has been considered to be the most comprehensive measure of dependence for evaluating total dependence, and several methods have been suggested for discerning the linear and nonlinear components of dependence between two variables. We, in this study, propose employing the Rényi and Tsallis mutual information measures for measuring total dependence because of their parametric nature. We first use a residual analysis in order to remove linear dependence between the variables, and then we compare the Rényi and Tsallis mutual information measures of the original data with that the lacking linear component to determine the degree of nonlinearity. A comparison against the values of the Shannon mutual information measure is also provided. Finally, we apply our method to the environmental Kuznets curve (EKC) and demonstrate the validity of the EKC hypothesis for Eastern Asian and Asia-Pacific countries.

## 1. Introduction

An analysis of the dependence between two or more random variables can be traced back to the late 19th century, beginning with the works of mathematicians such as Gauss and Laplace. Later, Galton created the concept of correlation, which enabled Pearson to derive the correlation coefficient that has been extensively used in all kinds of statistical analyses since then [1]. When the dependence is linear or approximately linear, the correlation coefficient is the most effective indicator of the relationship between the random variables. It also provides a simple interpretation for the direction of the relation, whether positive or negative. When the dependence departs from the linearity, the linear correlation coefficient is of no use, and various methods have been proposed for evaluating nonlinearity. One of these measures is Spearman’s correlation coefficient, which is nonparametric and uses ranked values to assess monotonic nonlinearity between two random variables [2]. Another measure for nonlinear dependence is the correlation ratio, which expresses the relationship between random variables as a single valued function. In the case of nonlinear relationships, the value of the correlation ratio is greater than the correlation coefficient, and therefore, the difference between the correlation ratio and the correlation coefficient refers to the degree of the nonlinearity of dependence [3]. Polynomial regression has also been used for modeling nonlinear dependence in various phenomena. Although nonparametric regression models have been used more often, polynomial regression is still being deployed for modeling dependence in some areas of application, such as biomechanics [4], cosmology [5], climatization [6], and chemistry [7]. As more and more-complex data have been produced through technological development, the need for analyzing these data have given rise to a new field, called functional data analysis, which also includes functional regression. Functional regression models assume functional relationships between responses and predictors, and for polynomial models, these relationships are in polynomial form rather than linear [8].

Shannon entropy plays a central role in information theory as a measure of information choice and uncertainty. Conditional entropy can also be used as a measure of missing information [9]. Conditional entropy or mutual information do not assume any underlying distribution and reflect the stochastic relationship between random variables as a whole—linear or nonlinear [10]. These properties have made mutual information a good choice for analyzing dependencies. Hence, mutual information is extensively used for dependency analysis, especially in finance [11,12,13] and in genetics [14,15,16]. Although mutual information is an effective method for determining the dependency between random variables, it does not provide any information on the nature of the dependence as being linear or nonlinear. Very few attempts have been made to investigate the nature of the dependence by extracting the linear component of Shannon mutual information, though some have, such as [1,17].

**Figure 1:**
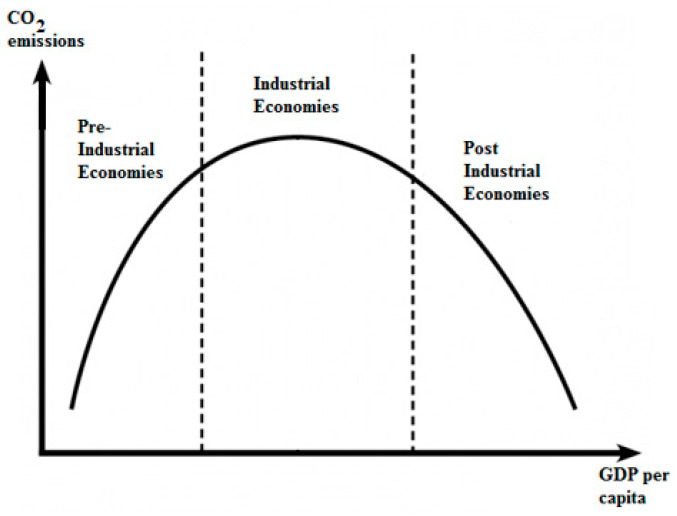
Environmental Kuznets curve.

The environmental Kuznets curve (EKC) hypothesis states that there is an inverse U-shape relationship between per capita gross domestic product (GDP) and measures of environmental degradation [18]. Because carbon dioxide ($CO_{2}$) is the major factor for greenhouse gas emissions, it is accepted as the main reason for the environmental degradation. Hence, the same relationship is assumed between GDP and $CO_{2}$. So the EKC is an indication of the “stages of economic growth” that economies pass through as they make a transition from agriculturally based to industrial and then to postindustrial service-based economies. In a way, EKC provides a visual representation of the stages of economic growth, as seen in Figure 1 (Panayatou 1993).

There are various methods in the literature to test the EKC. Some studies have used panel data, while others have used time series data [19]. Panayotou [20], who first suggested the term EKC, used cross-sectional data and empirically tested the relation between environmental degradation and economic development for the late 1980s. He discovered quadratic patterns in a sample of developing and developed countries. Antle and Heidebrink [21] found turning points for the EKC curve by using cross-sectional data. Vasilev [22] also studied EKC with cross-sectional data.

Although the determination of the exact shape of the Kuznets curve is important, demonstrating its nonlinearity will help support the EKC hypothesis. We aim to determine nonlinearity by deploying mutual information with an application on EKC. The Rényi and Tsallis mutual information types are used in determining the nonlinearity of EKC, and the results are compared with that of Shannon. By demonstrating the confirmation of the EKC hypothesis, it can be concluded that the “grow and pollute now, clean later” strategy revealed by the hypothesis has enormous environmental costs, so alternative strategies should be developed for growth.

The structure of our study is as follows: Section 2 describes the tests for nonlinearity on the basis of mutual information. Section 3 starts with the application by conducting a cross-sectional analysis using ordinary least squares (OLS) and then adds the application of nonlinearity tests. Finally, Section 4 concludes.

## 2. Relative Entropy, Mutual Information, and Dependence

### 2.1. Mutual Information

Relative entropy is a special case of statistical divergence. It is a measure of the inefficiency of assuming that the probability distribution is *q* when the true distribution is *p* [23]. Shannon, Rényi, and Tsallis relative entropies for the discrete case are defined as follows:

(1) $$D_{S}=(p\|q)=\sum P(x)\log\frac{P(x)}{q(x)}$$

(2) $$D_{R}=(p\|q)=\frac{1}{\alpha -1}\log{\sum P(x)\left(\frac{P(x)}{q(x)}\right)}^{\alpha -1}$$

(3) $$D_{T}=(p\|q)=\frac{1}{\alpha -1}\sum P(x)\left[{\left(\frac{P(x)}{q(x)}\right)}^{\alpha -1}-1\right]$$

Bivariate extensions are as follows:

(4) $$D_{S}(p(x,y)\|q(x,y))=\sum \sum p(x,y)\log\frac{p(x,y)}{q(x,y)}$$

(5) $$D_{R}(p(x,y)\|q(x,y))=\frac{1}{\alpha -1}\log{\sum \sum p(x,y)\left(\frac{p(x,y)}{q(x,y)}\right)}^{\alpha -1}$$

(6) $$D_{T}((p(x,y)\|q(x,y))=\frac{1}{\alpha -1}\sum \sum p(x,y)\left[{\left(\frac{p(x,y)}{q(x,y)}\right)}^{\alpha -1}-1\right]$$

To check the independence of variables, the null and alternative hypotheses can be stated as follows:

(7) $$H_{0}:p_{X,Y}(x,y)=q_{X,Y}(x,y)$$

(8) $$H_{A}:p_{X,Y}(x,y)\neq q_{X,Y}(x,y)$$

where $q_{X,Y}(x,y)=p_{X}(x)\cdot p_{Y}(y)$ for all $(x,y)\in R^{2}$.

Mutual information can be seen as the divergence of the joint probability function from the product of the two marginal probability distributions. In other words, mutual information is derived as a special case of divergence or relative entropy. Three alternative formulations of mutual information are due to Shannon, Rényi, and Tsallis. Shannon mutual information (or Kullback—Leibler divergence) is defined as follows:

(9) $$M(X,Y)=D_{S}\left(p_{X,Y}(x,y)\|p_{X}(x)p_{Y}(y)\right)=\sum \sum p(x,y)\log\frac{p(x,y)}{p_{X}(x)p_{Y}(y)}$$

Mutual information formulated this way is also called as cross entropy.

Rényi order-*α* divergence (or Rényi mutual information) of $p_{X,Y}(x,y)$ from $p_{X}(x)p_{Y}(y)$ is given as follows:

(10) $$D_{R}\left(p_{X,Y}(x,y)\|p_{X}(x)p_{Y}(y)\right)=\frac{1}{\alpha -1}\log\sum \sum \frac{p_{X,Y}{\left(x,y\right)}^{\alpha }}{{\left(p_{X}(x)p_{Y}(y)\right)}^{\alpha -1}}$$

Tsallis order-*α* divergence of $p_{X,Y}(x,y)$ from $p_{X}(x)p_{Y}(y)$ (or Tsallis mutual information) is given as follows:

(11) $$D_{T}\left(p_{X,Y}(x,y)\|p_{X}(x)p_{Y}(y)\right)=\frac{1-\sum \sum \frac{p_{X,Y}{\left(x,y\right)}^{\alpha }}{{\left(p_{X}(x)p_{Y}(y)\right)}^{\alpha -1}}}{1-\alpha }$$

In the case of independence, the Rényi and Tsallis mutual information types are 0, just like Shannon mutual information. As *α* → 1, the Rényi and Tsallis mutual information types approach Shannon mutual information [24]. The mutual information of two variables reflects the reduction in the variability of one variable, by knowing the other. Mutual information becomes 0 if and only if the random variables are independent. It should also be emphasized that mutual information measures general dependence, whereas the correlation coefficient measures linear dependence [15].

### 2.2. Testing Linearity by Using Mutual Information

The application of the Shannon mutual information measure on the problem of detecting nonlinearity was suggested by Tanaka, Okamoto, and Naito [17] and by Smith [1].

This method utilizes the residuals obtained by the ordinary linear regression model. Note that a linear regression model that fits data well is a good indicator of linear relation between variables so that the residuals obtained from a linear model are considered to include no linear dependence on independent variables:

(12) $$\xi_{i}=Y_{i}-b_{0}-\sum_{j=1}^{p}b_{j}X_{j}$$

Next, the mutual information between residuals and observed values of the independent variable is calculated. The mutual information between independent and dependent variables *M*(*X*,*Y*) can be computed, as can the mutual information between independent variable and the residuals obtained from linear regression *M*(*X*,*ξ*). Note that the later statistic reflects the nonlinear dependence between the original variables. If the mutual information between the independent variable and residuals does not differ much from the mutual information between the dependent and independent variables, then the relation is nonlinear. By comparing *M*(*X*,*ξ*) with *M*(*X*,*Y*), we can evaluate the degree of nonlinearity in the dependence [1,17].

We suggest that nonlinearity can be detected better by the Rényi and Tsallis mutual information measures because of their parametric nature.

Especially becauase the Tsallis mutual information measure is calculated on the basis of the power of *α*, the larger the *α* value, the larger the Tsallis mutual information was becoming, so the difference between these two common mutual information measures cannot be interpreted. Therefore, we suggest a new measure that still leads to the same result, as seen in Equation (13):

(13) $$\lambda_{S,R,T}=\left|1-\frac{M(X,\xi )}{M(X,Y)}\right|$$

The letters *S*, *R*, and *T* in the index indicate the Shannon, Rényi, and Tsallis mutual information measures, respectively. As *M*(*X*,*ξ*) and *M*(*X*,*Y*) become closer to each other, *λ* converges to zero, implying nonlinearity. This hypothesis is tested by using two simulated data sets, one of which represents a linear relationship and the other one reflects curvilinearity. The number of simulated pairs of *X* and *Y* values is 1000. The simulated data representing the linear and the curvilinear relationships are modeled by Equations (14) and (15):

(14) Y=a+bX+e

(15) $$Y=a+bX+cX^{2}+e$$

Various *α* values between 0 and 5 are selected randomly from a uniform distribution for assessing the effect of *α* on nonlinearity measures. Table 1 provides 50 randomly generated observations from a uniform distribution for different values of *α* and the corresponding *λ* values for the Rényi and Tsallis measures.

Because *λ* values close to 1 indicate a linear relationship, $\lambda_{T}$, $\lambda_{S}$, and $\lambda_{R}$ support the linearity hypothesis. It can be observed that $\lambda_{T}$ detects linearity more strongly than does $\lambda_{R}$ for *α* > 1; conversely, $\lambda_{R}$ captures linearity better for *α* < 1. The mean and the standard deviation for each mutual information measure are also presented in Table 1 for checking the variability of each measure against various *α* values. The standard deviation values for $\lambda_{T}$ are lower than those for $\lambda_{R}$, pointing out the consistency of Tsallis in the case of linearity.

On the other hand, nonlinearity is captured by $\lambda_{T}$ better than by $\lambda_{R}$ for *α* < 1 and vice versa for *α* > 1. When the standard deviations are considered, $\lambda_{R}$ is more stable in determining nonlinearity.

**Figure 2:**
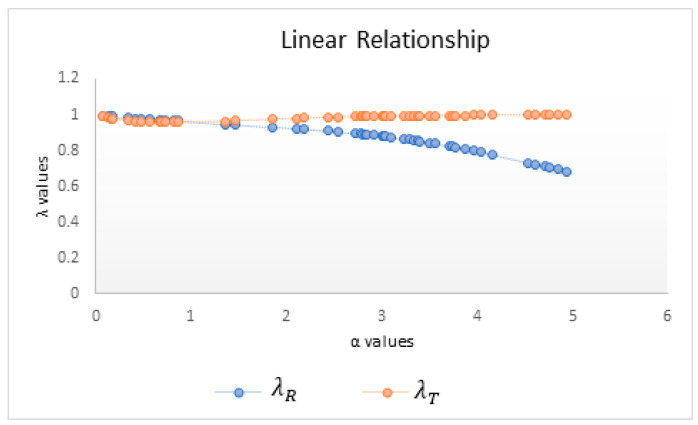
*λ* values versus *α* in the case of linearity.

Changing the scale parameter *α* of mutual information measures naturally changes the sensitivity of this measure, and by plotting *λ* values against the scale parameter *α*, the change in sensitivity can be graphically displayed. In order to visually interpret the results, the $\lambda_{R}$ and $\lambda_{T}$ values, according to the different *α* values, are as seen in the graphs:

As can be seen from Figure 2, for *α* > 1, $\lambda_{T}$ is more succesful and stable than $\lambda_{R}$ for a linear relationship. In addition, the $\lambda_{T}$ measure consistently takes values close to 1, whereas $\lambda_{R}$ gets smaller as *α* values increase.

**Figure 3:**
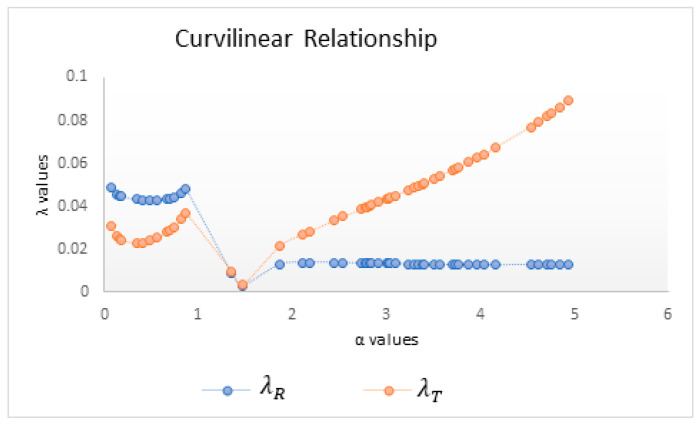
*λ* values versus *α* in the case of curvilinearity.

As seen in Figure 3, in the curvilinear relationship, $\lambda_{T}$ started to grow after alpha 1.4; $\lambda_{R}$ takes values close to zero in all values of alpha. However, $\lambda_{T}$ also takes a maximum value of 0.09101. Both common information measures can be used as criteria in nonlinearity. However, $\lambda_{R}$ more consistently indicates nonlinearity. Because there is no logarithmic function in the Tsallis mutual information formula, when *α* takes a value greater than 1, Tsallis mutual information makes deviations from linearity less important than Rényi mutual information does. Therefore, $\lambda_{T}$ will make it less sensitive to nonlinearity than $\lambda_{R}$ and therefore more unresponsive to nonlinearity than $\lambda_{S}$ and $\lambda_{T}$. For the same reason, $\lambda_{T}$ will represent linearity better than Rényi will in linear relationship.

An important general property of Rényi entropy is that for a given probability distribution, Rényi entropy is a monotonically decreasing function of *α*, where *α* is an arbitrary real number other than 1. Therefore, as can be seen in Figure 2, increasing *α* values will not provide additional information, so *α* values are limited to 5.

### 2.3. Method for Bin-Size Selection

Mutual information depends mainly on both the bin size and the sample size; thus, a natural question arises about the optimal choice of one parameter given the value of another. Here, we use the Freedman–Diaconis rule for finding the optimal number of bins. According to this rule, the optimal number of bins can be calculated on the basis of the interquartile range ($IQR=Q_{3}-Q_{1}$) and the number of data points *n*. Freedman and Diaconis use the IQR of the data instead of the standard deviation; therefore, this method is described as more robust than some of the other methods.

(16) $$\Delta_{bin}=2\times \frac{IQR\left(X\right)}{\sqrt[{3}]{n}}$$

The Freedman–Diaconis rule takes into account the asymmetry of the data and sets the bin size to be proportional to the IQR [25].

## 3. Checking the EKC Hypothesis for East Asian and Asia-Pacific Countries (1971–2016)

### 3.1. Model

To test the EKC hypothesis, a simple linear regression model is applied. Using the ordinary least squares procedure, we find a quadratic relationship (“inverted U-hypothesis”) between $CO_{2}$ emissions (metric tons per capita) and GDP per capita (current USD) in a time series of East Asia and Asia-Pacific countries (excluding high-income countries) over a 46-year period.

East Asia and Asia-Pacific countries were classified initially as low income (LIC) in the 1990s, then as lower middle income (LMC) in 2010. In fact, the highest growth rate of $CO_{2}$ emissions (5.6% (1990–2008)) was observed in the East Asia and the Asia-Pacific region, where the highest GDP growth rates (7.2% (1990–2000) and 9.4% (2000–2010)) were achieved.

**Figure 4:**
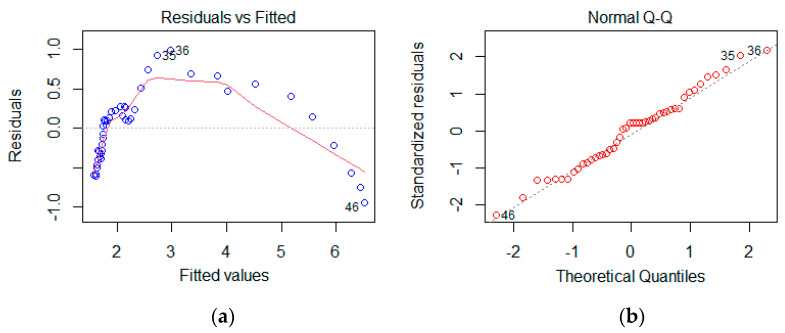
Residual plots listed as (**a**) fitted values to residuals and (**b**) normal Q-Q plot of standardized residuals.

**Figure 5:**
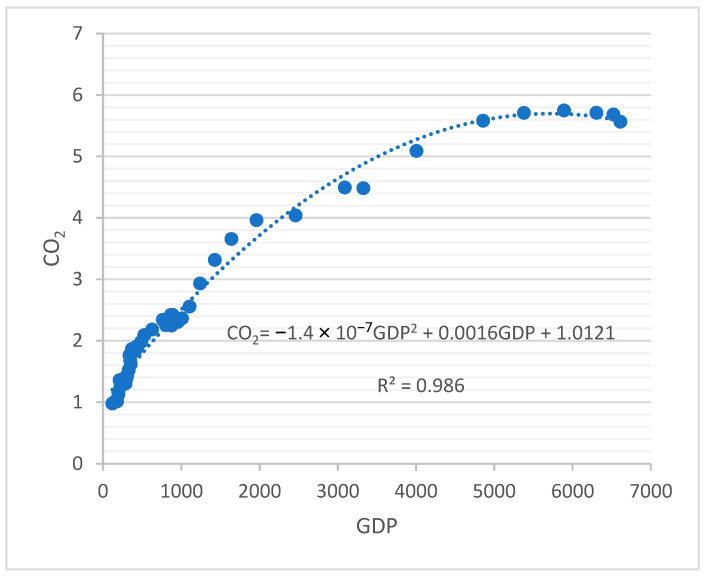
Quadratic regression model estimation.

We first examine the residual diagrams from a linear regression model to determine whether there are serious deviations from assumptions. In Figure 4a, nonlinearity is apparent, whereas in Figure 4b, the deviation from normality assumption can be seen:

According to a quick visual check of the residuals in Figure 4a, a quadratic model seems to be more appropriate. In Table 2, the results of quadratic models are given. The scatter diagram of $CO_{2}$ and GDP variables is shown in Figure 5.

To test the appropriateness of a simple linear regression function, the null and alternative hypotheses are given as follows:

(17) $$H_{0}:E(Y)=\beta_{0}+\beta_{1}X$$

(18) $$H_{a}:E(Y)\neq \beta_{0}+\beta_{1}X$$

The general linear test statistic for simple regression model is as follows:

$$F^{*}=\frac{SSLF}{c-2}÷\frac{SSPE}{n-c}=\frac{1.641}{0.107}=15.209$$

When we look at the results, shown in Table 3, F*>F(0.05;3.41)=2.833, so we reject null hypothesis $H_{0}$. This means that the linear regression function does not provide a good fit for the data. The dependence measures are $r^{2}=0.91$ and $\eta_{XY}^{2}=0.96$. A nonzero value of $\eta_{YX}^{2}-r^{2}$ is associated with a departure from linearity. The calculated value of this difference is $\eta_{YX}^{2}-r^{2}=0.05$. To test the significance of this difference, the alternatives are given as follows:

$H_{0}$: The relationship between *X* and *Y* is linear.

$H_{a}$: The relationship between *X* and *Y* is not linear.

The test statistic is as follows:

$$F^{*}=\frac{n-c}{c-2}\cdot \frac{\eta_{XY}^{2}-r^{2}}{1-\eta_{XY}^{2}}=\frac{41}{3}*\frac{0.05}{0.04}=17.083$$

This value of F also indicates a significant departure from linearity.

### 3.2. Testing Linearity on the Basis of Shannon, Rényi, and Tsallis Mutual Information Measures

The Tanaka, Okamoto, and Naito [17] and Smith [1] method is based on comparing the Shannon mutual information between the original data series with that between the new ones obtained by removing linear dependence from the original ones.

Entropy and mutual information calculations are based on a contingency table. A possible reason for the EKC hypothesis may lie in the fact that in poor countries, most of the output is produced in the agricultural sector. So $CO_{2}$ emissions are lower in these countries than in other countries. In middle-income countries, pollution begins to increase. As the country grows, it tends to switch to cleaner technologies.

Here, on the basis of the Freedman–Diaconis rule, the optimal number of bins is calculated and presented in Table 4:

To detect nonlinearity by using the Shannon, Rényi and Tsallis mutual information measures, the following table for different values of alpha may help. To evaluate the degree of nonlinearity included in the dependence, the two mutual information measures were compared. When M(X,ξ)=M(X,Y), the dependence is interpreted to be based on nonlinearity, so the proposed $\lambda_{S}$, $\lambda_{R}$, and $\lambda_{T}$ measures are considered as criteria of nonlinearity.

As seen in the Table 5, $\lambda_{S}$, $\lambda_{R}$, and $\lambda_{T}$ are close to zero, so the relationship is nonlinear. As can be checked from the simulation data in Table 1, *α* < 1 $\lambda_{T}$ and *α* > 1 $\lambda_{R}$ more successfully reveal the curvature. Therefore, the results obtained from the EKC data also support this situation. As a result, the $\lambda_{S}$, $\lambda_{R}$, and $\lambda_{T}$ values nearly zero indicate a curvilinear relationship, which supports the EKC hypothesis.

**Figure 6:**
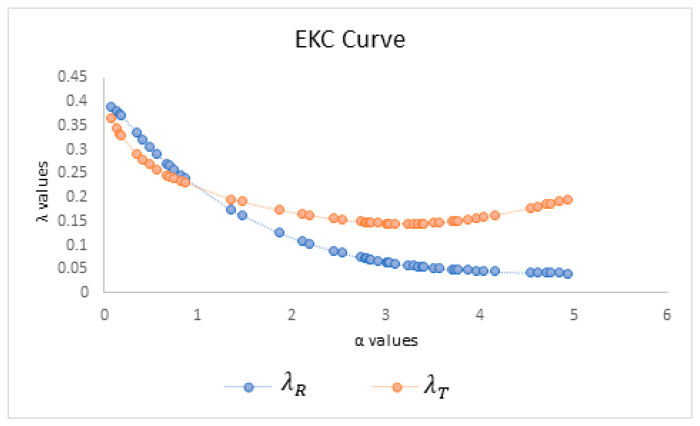
*λ* values against different *α* values for EKC data.

The relationship between *λ* and *α* can be seen in Figure 6:

## 4. Conclusions

The environmental Kuznets curve (EKC) hypothesizes that the relationship between environmental quality and real output has an inverted U-shaped quality. Using the ordinary least squares estimation procedure, we have found a quadratic relationship between $CO_{2}$ emission and GDP in a time series of East Asia and Asia-Pasific countries (excluding high-income countries) over a period of 46 years. One technique to check the EKC hypothesis utilizes an F test, by which we have concluded that the linear model does not provide a good fit for the data. As a second technique, comparing the linear determination coefficient with the correlation ratio may be useful. Again, for the EKC data, the difference between these two association measures was found to be significant, addressing curvilinearity. Alternatively, the difference between two dependence measures on the basis of mutual information can be used. Although Shannon mutual information has been used more often in the literature, we suggested that the Rényi and Tsallis mutual information measures catch the nature of the relation between the variables better because of their parametric flexibility.

In this study, the mutual information between dependent and independent variables (*M*(*X*,*Y*)) was found first. Secondly, by using a simple linear regression model, the residuals (*ξ*) were calculated. Then, the mutual information between the independent variable and the residuals (*M*(*X*,*ξ*)) was obtained. Finally, by comparing these two mutual information measures, the degree of nonlinearity included in the dependence was determined. We also proposed a measure of nonlinearity, *λ*, and demonstrated that the Rényi and Tsallis mutual information measures determined nonlinearity better for certain ranges of *α* values compared with the Shannon mutual information measure.

Applications of all these measures on $CO_{2}$ emissions and GDP data underlined curvilinearity, and hence, the presumed pattern by the EKC hypothesis was realistic. The result concludes that the “growth and pollute now, clean later” strategy is wasting a lot of resources and has enormous environmental costs. Therefore, countries should seek alternative growth strategies.

## Figures and Tables

**Table 1:** *λ* values for linear and curvilinear relationships, based on simulations.

	Linear Relationship		Curvilinear Relationship	
*α*	*λ_R_*	*λ_T_*	*λ_R_*	*λ_T_*
0.07	0.9956	0.9906	0.0489	0.0308
0.13	0.9917	0.983	0.0457	0.0263
0.17	0.9894	0.9788	0.0447	0.0247
0.18	0.9888	0.9778	0.0445	0.0244
0.35	0.9808	0.9664	0.0433	0.0228
0.41	0.9785	0.964	0.0431	0.0232
0.48	0.976	0.9619	0.0429	0.024
0.56	0.9734	0.9604	0.0429	0.0254
0.67	0.9702	0.9595	0.0435	0.0281
0.69	0.9696	0.9595	0.0437	0.0287
0.74	0.9684	0.9596	0.0444	0.0304
0.82	0.9668	0.9605	0.0464	0.0339
0.87	0.9663	0.9618	0.0483	0.0369
1.36	0.9473	0.964	0.0087	0.0099
1.46	0.9446	0.9662	0.003	0.0037
1.86	0.9323	0.9749	0.0129	0.0214
2.11	0.9236	0.9797	0.0138	0.0271
2.18	0.9209	0.981	0.0139	0.0285
2.44	0.9102	0.9851	0.0139	0.0334
2.54	0.9056	0.9865	0.0138	0.0352
2.73	0.8962	0.9888	0.0136	0.0386
2.78	0.8935	0.9893	0.0136	0.0395
2.8	0.8924	0.9895	0.0136	0.0398
2.83	0.8908	0.9898	0.0135	0.0403
2.84	0.8902	0.9899	0.0135	0.0405
2.92	0.8856	0.9907	0.0134	0.0419
3.01	0.8801	0.9914	0.0133	0.0436
3.02	0.8795	0.9915	0.0133	0.0437
3.04	0.8782	0.9917	0.0133	0.0441
3.09	0.8749	0.9921	0.0132	0.045
3.23	0.8652	0.9931	0.0131	0.0476
3.29	0.8608	0.9934	0.0131	0.0487
3.34	0.857	0.9937	0.013	0.0497
3.38	0.8538	0.994	0.013	0.0504
3.4	0.8522	0.9941	0.013	0.0508
3.5	0.844	0.9946	0.0129	0.0528
3.57	0.8379	0.9949	0.0128	0.0542
3.71	0.8249	0.9954	0.0128	0.057
3.74	0.822	0.9956	0.0128	0.0576
3.77	0.8191	0.9957	0.0127	0.0583
3.88	0.808	0.996	0.0127	0.0607
3.97	0.7985	0.9963	0.0127	0.0627
4.04	0.7908	0.9964	0.0127	0.0643
4.17	0.7762	0.9967	0.0127	0.0673
4.54	0.7318	0.9974	0.0128	0.0769
4.62	0.7219	0.9975	0.0128	0.0792
4.71	0.7108	0.9976	0.0129	0.0818
4.76	0.7046	0.9976	0.0129	0.0833
4.85	0.6934	0.9977	0.013	0.0861
4.94	0.6822	0.9978	0.0131	0.089
$\lambda_{S}$	0.9589		0.0121	
Mean	0.8783	0.9848	0.0211	0.0443
Std. Dev.	0.0869	0.0134	0.0143	0.0199

**Table 2:** Summary of the model.

	a	b	c	F	$R^{2}$
Parameter Estimates	1.0121	0.0016	1.4 × 10^−7^	1581.224	0.986
Standard Error	0.04599	5.9 × 10^−5^	9.35 × 10^−9^		
*p*-Value	4.99 × 10^−25^	4.39 × 10^−29^	5.08 × 10^−19^		
Model	$CO_{2}=1.021+0.0016GDP-\left(1.4\times 10^{-7}\right)GDP^{2}$				

a: constant; b and c: coefficients of model; F: F test; R^2^: coefficient of determination.

**Table 3:** Related ANOVA table.

Source of Variation	Df	Sum of Squares	Mean Squares
Explained variation by linear regression	1	SSR = 98.658	98.658
Explained variation by nonlinear regression	3	SSLF = 4.925	1.641
Unexplained variation	41	SSPE = 4.425	0.107
Total	45	SST = 108.01	

**Table 4:** Optimal number of bins.

Variables	$n_{bins}$
$CO_{2}$	7
GDP	14
Residuals	9

**Table 5:** *λ* values for EKC data.

*α*	*λ_R_*	*λ_T_*	*α*	*λ_R_*	*λ_T_*
0.07	0.3892	0.3655	3.01	0.0649	0.1460
0.13	0.3809	0.3444	3.02	0.0646	0.1459
0.17	0.3741	0.3323	3.04	0.0640	0.1458
0.18	0.3722	0.3295	3.09	0.0625	0.1455
0.35	0.3355	0.2902	3.23	0.0587	0.1453
0.41	0.3221	0.2797	3.29	0.0573	0.1455
0.48	0.3071	0.2690	3.34	0.0562	0.1457
0.56	0.2910	0.2586	3.38	0.0553	0.1460
0.67	0.2708	0.2466	3.4	0.0549	0.1461
0.69	0.2673	0.2447	3.5	0.0530	0.1471
0.74	0.2590	0.2402	3.57	0.0518	0.1481
0.82	0.2468	0.2339	3.71	0.0497	0.1505
0.87	0.2400	0.2307	3.74	0.0493	0.1511
1.36	0.1735	0.1961	3.77	0.0489	0.1518
1.46	0.1631	0.1913	3.88	0.0476	0.1545
1.86	0.1271	0.1743	3.97	0.0467	0.1570
2.11	0.1087	0.1653	4.04	0.0461	0.1591
2.18	0.1040	0.1630	4.17	0.0451	0.1635
2.44	0.0888	0.1556	4.54	0.0430	0.1782
2.54	0.0837	0.1532	4.62	0.0427	0.1817
2.73	0.0751	0.1494	4.71	0.0423	0.1858
2.78	0.0731	0.1486	4.76	0.0421	0.1881
2.8	0.0723	0.1483	4.85	0.0418	0.1923
2.83	0.0711	0.1479	4.94	0.0415	0.1965
2.84	0.0708	0.1477	*λ_S_*	0.2181	0.2181
2.92	0.0679	0.1468	Mean	0.1313	0.1914
			St. Dev.	0.1147	0.0607

## Data Availability

Data given within manuscript.
